# Knockdown of Sly-miR164a Enhanced Plant Salt Tolerance and Improved Preharvest and Postharvest Fruit Nutrition of Tomato

**DOI:** 10.3390/ijms24054639

**Published:** 2023-02-27

**Authors:** Xue Wan, Zhiqiang Wang, Wenhui Duan, Taishan Huang, Hongmiao Song, Xiangbin Xu

**Affiliations:** Key Laboratory of Food Nutrition and Functional Food of Hainan Province, School of Food Science and Engineering, Hainan University, Haikou 570228, China

**Keywords:** tomato, miR164a, salt tolerance, fruit nutrition

## Abstract

Salinity stress is a serious limitation to tomato growth and development. The aim of this study was to investigate the effects of Sly-miR164a on tomato growth and fruit nutritional quality under salt stress. The results showed that the root length, fresh weight, plant height, stem diameter and ABA content of miR164a#STTM (knockdown of Sly-miR164a) lines were higher than those of WT and miR164a#OE (overexpression of Sly-miR164a) lines under salt stress. Compared with WT, miR164a#STTM tomato lines exhibited lower ROS accumulation under salt stress. In addition, the fruits of miR164a#STTM tomato lines had higher soluble solids, lycopene, ascorbic acid (ASA) and carotenoid content compared with WT. The study indicated that tomato plants were more sensitive to salt when Sly-miR164a was overexpressed, while knockdown of Sly-miR164a enhanced plant salt tolerance and improved fruit nutritional value.

## 1. Introduction

Tomato is an immensely popular food, which is rich in ASA, lycopene, carotenoids, and other beneficial active components, and also is one of the main cash crops in the world [1]. However, adverse environmental conditions during their life cycle, such as salinity, drought and extreme temperatures, can pose a serious threat to tomato seed germination, plant growth and fruit yield [2]. Among them, salt stress induces osmotic stress by reducing soil water potential and water supply, which produces excess toxic reactive oxygen species (ROS) and malondialdehyde (MAD), affecting crop physiological and biochemical pathways, inhibiting crop growth and reducing yields [3,4]. Plants have established various biochemical, physiological and molecular mechanisms to respond to salt stress rapidly [5].

MicroRNAs (miRNAs) are small endogenous non-coding RNAs with a length of 18–24 nt and widely found in plants [6,7,8]. The miRNAs perform a critical role in abiotic responses by regulating levels of transcription factors [9]. During salt stress, miRNAs and the transcription factors of their targets are involved in regulating key metabolic processes in plants [10]. In the salt-tolerant maize line ‘NC286’ and the salt-sensitive maize line ‘Huangzao4’, the miR156, miR164, miR167 and miR396 families downregulated in response to salt stress, and the salt-responsive miRNA was involved in the regulation of metabolism and physiological adaptation in maize seedlings at the post-transcriptional level, indicating that the miRNA expression model was related to salt sensitivity [11]. Under salt stress, overexpression of *Osa-miR393* in *Arabidopsis thaliana* and rice led to a decrease in growth rate and salt tolerance [12]. Liu et al. [13] reported that with overexpressed miR396a in *chrysanthemum*, the content of proline in leaves increased, and the salt tolerance level increased. Meanwhile, Shan et al. [14] found that down-regulation of Zma-miR164 resulted in the increased expression levels of target genes (*GRMZM2G114850* and *GRMZM2G008819*), which significantly enhanced salt tolerance of maize. These studies suggested that miRNAs mediate target genes to regulate abiotic stress in plants. Therefore, the function of miRNAs under salt stress is of great significance to the sustainable production of tomatoes [5]. 

The miR164 family is a plant-specific group of miRNAs that directionally regulates the NAC-domain gene [15,16,17,18]. The NAC domain transcription factor is plant-specific, the target of miR164, which has been shown to regulate the development of fruit and respond to abiotic and biotic stresses [19,20]. The expression of miR164 was up-regulated in response to osmotic stress, and the target gene *NAC1* was down-regulated in plant salt stress [21]. During the salt stress period, stress genes were up-regulated in *SNAC1* overexpressing rice plants, significantly improving rice sensitivity to abscisic acid [22]. Understanding the function of miR164 could have great significance for improving tomato stress tolerance.

In this study, the role of miR164a in the salt tolerance of tomato was investigated. The expression pattern of miR164 under salt stress was analyzed and its effects on plant growth and fruit quality were tested. New insights were provided for miR164a-mediated responses to salt stress in tomato.

## 2. Results

### 2.1. Knockdown of miR164a Promotes Germination of Transgenic Plant Seeds under Salt Stress

**Figure 1:**
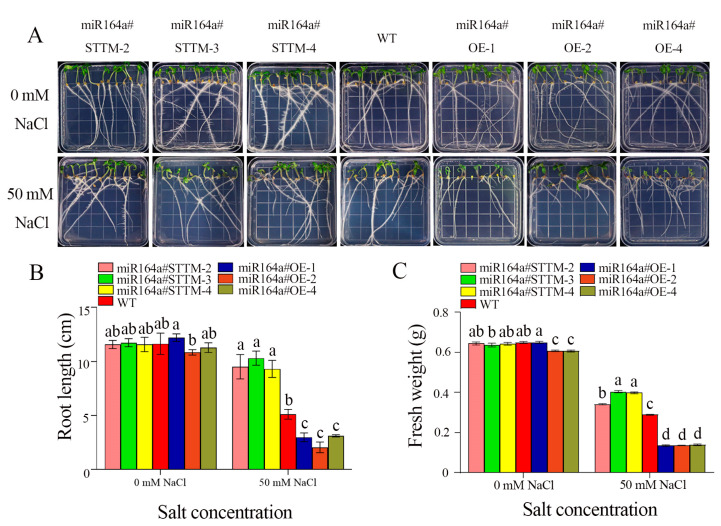
Comparative analysis of miR164a#STTM, WT and miR164a#OE tomato seed germination under salt stress. (**A**) Germination of miR164a#STTM, WT and miR164a#OE tomato seed in a medium containing 50 mM NaCl. (**B**) Root length of miR164a#STTM, WT and miR164a#OE tomato seed under salt treatment. (**C**) Fresh weight of miR164a#STTM, WT and miR164a#OE tomato seedlings under salt treatment. Vertical bars represent standard deviations of the means, *n* = 3. Different letters indicated significant differences between WT and transgenic tomatoes in the values at *p* < 0.05.

As shown in Figure 1A, there were no significant differences in the growth phenotypes of miR164a#STTM, miR164a#OE and WT lines grown in 0 mM NaCl ½ × MS medium after 14 days. In the presence of 50 mM NaCl, the growth of miR164a#STTM and WT plants was less adversely affected by salinity, but the growth of miR164a#OE transgenic plants was severely affected by salinity. Compared with miR164a#STTM and WT plants, salt stress significantly inhibited the growth of miR164a#OE tomato roots, and the leaves of miR164a#OE-1 showed severe wilting and the plants became smaller. After 14 days of salt stress, the average root lengths of miR164a#STTM, WT and miR164a#OE lines were 9.1, 4.82 and 2.61 cm, respectively (Figure 1B). The average fresh weight of miR164a#STTM was 0.38 g, whereas the average fresh weight of miR164a#OE lines and WT was 0.14 and 0.29 g, respectively (Figure 1C). Compared with WT, the fresh weight of miR164a#STTM lines was 1.11 times that of WT, and the fresh weight of miR164a#OE lines was 0.47 times that of WT. Compared with the WT, the root length and fresh weight measurements showed that the miR164a#OE line was more severely affected by salt stress, but the miR164a#STTM line showed better salt tolerance.

### 2.2. Knockdown of miR164a Alleviates ROS Accumulation in Transgenic Plants

**Figure 2:**
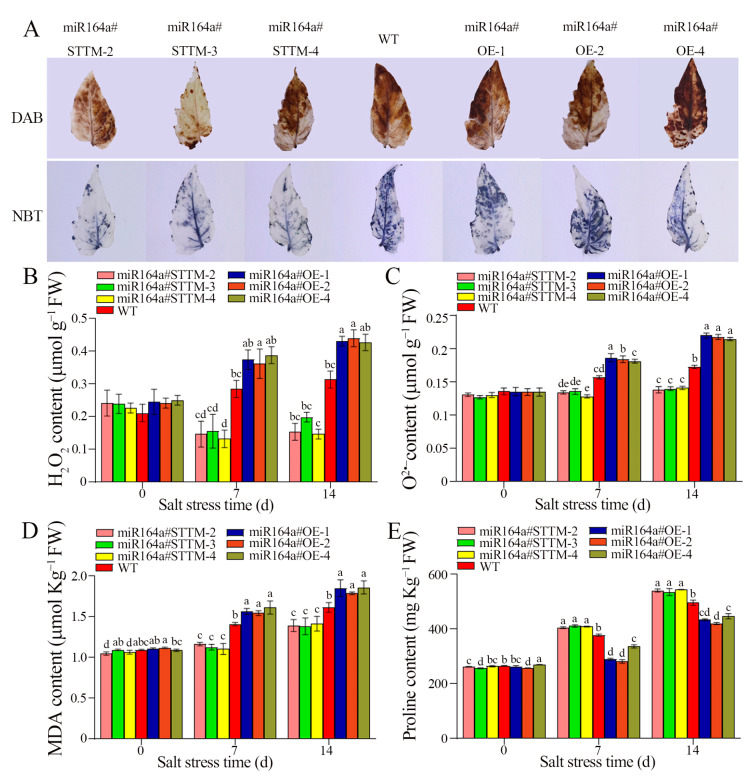
Analysis of ROS accumulation in miR164a#STTM, WT and miR164a#OE tomato seedlings under salt stress. (**A**) Comparison of DAB staining and NBT staining of miR164a#STTM, WT and miR164a#OE tomato leaf after 14 days of salt treatment. The H_2_O_2_ content (**B**), O^2•–^ content (**C**), MDA content (**D**) and proline content (**E**) in tomato seedling root. Vertical bars represent standard deviations of the means, *n* = 3. Different letters indicated significant differences between WT and transgenic tomatoes in the values at *p* < 0.05.

As shown in Figure 2A, there was accumulation of dark-brown 3,3-diaminobenzidine (DAB) polymerization and blue nitroblue tetrazolium (NBT) polymerization products in miR164a#STTM, miR164a#OE and WT leaves after 14 days treatment with 100 mM NaCl, but the leaves of miR164a#OE lines exhibited darker staining than WT leaves, and the leaves of miR164a#STTM lines exhibited lighter staining than WT leaves. After treatment with 100 mM NaCl for 14 days, the H_2_O_2_ contents in the roots of miR164a#STTM, WT and miR164a#OE lines were 0.18, 0.25, 0.47 μmol g*^−^*^1^, respectively (Figure 2B). The O^2•–^ contents in the roots of miR164a#STTM, WT and miR164a#OE lines were 0.14, 0.19, 0.22 μmol g*^−^*^1^, respectively (Figure 2C). The measurement of H_2_O_2_ and O^2•–^ contents showed that miR164a#OE lines exhibited higher accumulation of H_2_O_2_ and O^2•–^ than WT, while miR164a#STTM lines exhibited lower accumulation of H_2_O_2_ and O^2•–^ than WT.

After 14 days of salt stress, the average contents of malondialdehyde (MDA) in roots of miR164a#STTM, WT and miR164a#OE lines were 1.50, 1.93 and 2.03 μmol Kg*^−^*^1^, respectively (Figure 2D). Compared with WT, the contents of MDA in the roots of miR164a#STTM were lower than in the WT, and the contents of MDA in the roots of miR164a#OE were higher than in the WT. The average contents of proline in the roots of miR164a#STTM, WT and miR164a#OE lines were about 531.72, 495.86 and 439.42 mg Kg*^−^*^1^ (Figure 2E). The average content of proline in the roots of miR164a#STTM was relatively higher than that of WT, while in miR164a#OE it was relatively lower than that of WT.

### 2.3. Knockdown of miR164a Enhances Salt Tolerance in Transgenic Plants

**Figure 3:**
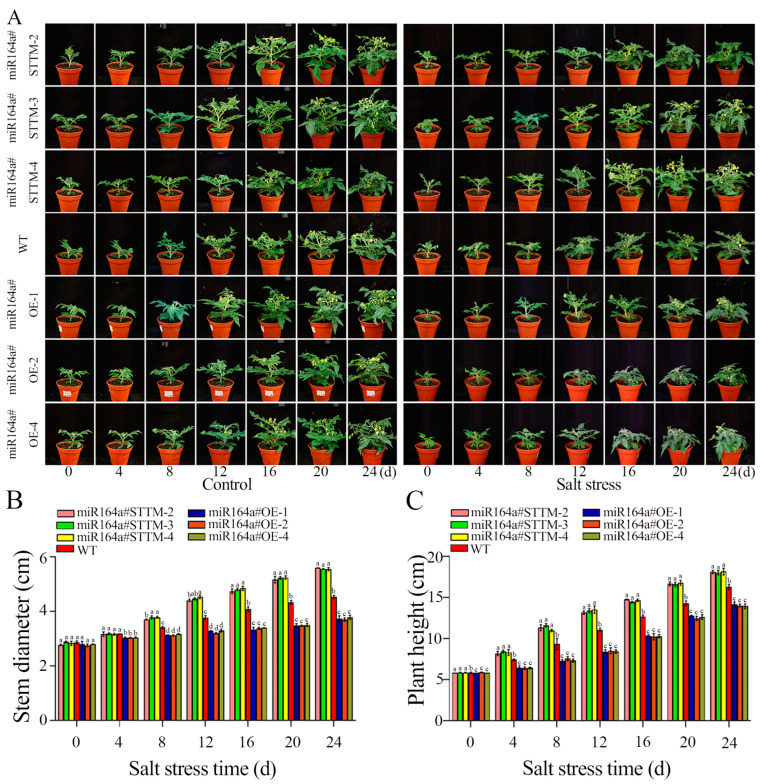
The growth of miR164a#STTM, WT and miR164a#OE tomato seedlings under control and salt stress. (**A**) Growth of miR164a#STTM, WT and miR164a#OE tomato seedlings under salt treatment for 24 days. (**B**) Stem diameter variation of miR164a#STTM, WT and miR164a#OE tomato seedlings under salt treatment. (**C**) Plant height variation of miR164a#STTM, WT and miR164a#OE tomato seedlings under salt treatment. Vertical bars represent standard deviations of the means, *n* = 3. Different letters indicated significant differences between WT and transgenic tomatoes in the values at *p* < 0.05.

As shown in Figure 3A, there were no obvious differences between miR164a#STTM, WT and miR164a#OE in terms of plant height and growth rate under control conditions. After 24 days of salt stress treatment, miR164a#STTM transgenic seedlings were significantly higher than WT, while miR164a#OE transgenic seedlings were significantly shorter. After 24 days of salt stress, the average plant height of miR164a#STTM-2, miR164a#STTM-3, miR164a#STTM-4 and WT were 18.06, 18.00, 18.13 and 16.23 cm, respectively. However, the average plant height of miR164a#OE-1, miR164a#OE-2 and miR164a#OE-4 only reached 14.13, 14.00 and 13.9 cm, respectively (Figure 3C). In terms of plant height, the miR164a#STTM lines were slightly taller than WT, while the miR164a#OE were significantly lower than WT. The average stem diameter of miR164a#STTM-2, miR164a#STTM-3, miR164a#STTM-4, WT, miR164a#OE-1, miR164a#OE-2 and miR164a#OE-4 reached 5.59, 5.55, 5.54, 4.52, 3.73, 3.69, and 3.77 cm, respectively. From the perspective of stem diameter, the average stem diameter of miR164a#STTM lines was 1.23 times thicker than that of WT, and the average stem diameter of miR164a#OE lines was 0.69 times thinner than that of WT (Figure 3B).

### 2.4. Fruit Quality Evaluation

**Figure 4:**
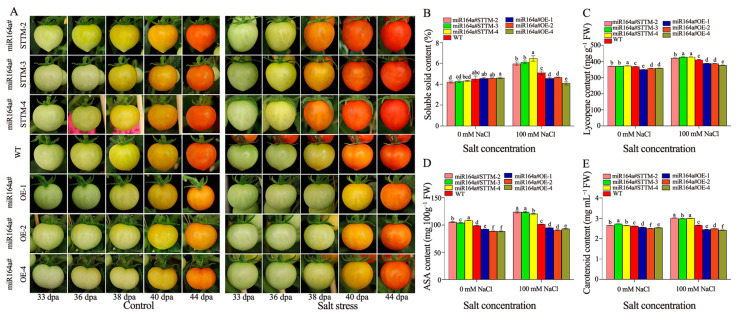
Changes of phenotypic characterization and nutritional quality of preharvest fruit in miR164a#STTM, WT and miR164a#OE tomato under control and salt stress. (**A**) Comparison of preharvest fruit development of miR164a#STTM, WT and miR164a#OE at 33–44 dpa under control and salt treatment. The contents of soluble solids (**B**), lycopene (**C**), ASA (**D**) and carotenoids (**E**) in miR164a#STTM, WT and miR164a#OE tomato fruit during salt treatment. Vertical bars represent standard deviations of the means, *n* = 3. Different letters indicated significant differences between WT and transgenic tomatoes in the values at *p* < 0.05.

As shown in Figure 4A, under control treatment, WT and miR164a#STTM fruits began to turn yellow at 36 dpa, while miR164a#OE fruits showed no color change; preharvest fruit discoloration in WT and miR164a#STTM transgenic tomatoes occurred 2 days earlier than in miR164a#OE tomatoes. Fruit discoloration was accelerated by salt stress treatment in comparison to control fruits. Under salt stress treatments, preharvest fruit discoloration in miR164a#STTM transgenic tomatoes occurred 2–3 days earlier than in WT and miR164a#OE tomatoes. Under salt stress treatment, miR164a#STTM fruits began to turn yellow at 36 dpa, while WT and miR164a#OE fruits showed no color change and remained at the mature green stage at that time. Under salt stress treatment, WT and miR164a#STTM tomato fruits were at the red ripening stage at 40 dpa, while miR164a#OE tomato fruits were only at the yellow ripening stage at that time. Under salt stress, the content of soluble solids in miR164a#STTM and WT tomato fruits increased to varying degrees. The content of soluble solids in miR164a#STTM-2, miR164a#STTM-3, miR164a#STTM-4 and WT fruits increased by 16.6%, 16.7%, 27.9% and 6.7% compared with the control fruits, respectively. The soluble solids content of miR164a#STTM-4 transgenic tomato fruit showed the highest increase under salt stress (Figure 4B). Under 100 mM NaCl stress, the average lycopene content in miR164a#STTM, WT and miR164a#OE tomato fruits were 425.87, 411.16 and 384.53 mg g*^−^*^1^ FW, respectively. The lycopene content of miR164a#STTM fruit was 1.04 times higher than that of WT, and miR164a#OE is 0.94 times that of WT under salt stress (Figure 4C). The average ASA contents in miR164a#STTM, WT and miR164a#OE tomato fruits with salt stress were 122.76, 101.33 and 92.99 mg 100 g*^−^*^1^ FW. The ASA content of miR164a#STTM fruits with salt stress was 1.21 times higher than that of WT, and miR164a#OE is 0.92 times that of WT (Figure 4D). The average carotenoid contents in miR164a#STTM, WT and miR164a#OE tomato fruits with salt stress were 3.00, 2.66 and 2.46 mg mL*^−^*^1^ FW, respectively. The carotenoid content of miR164a#STTM fruit with salt stress was 1.13 times that of WT, and miR164a#OE was 0.92 times that of WT (Figure 4E).

**Figure 5:**
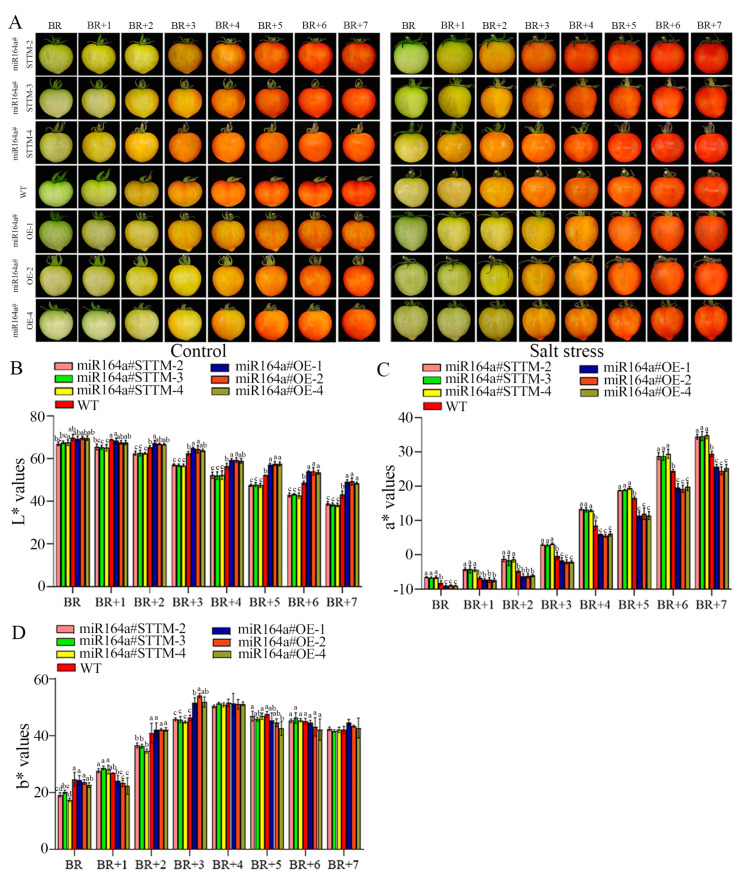
Phenotypes assessment of postharvest fruit of miR164a#STTM, WT and miR164a#OE tomato fruits under control and salt stress. (**A**) Comparison of postharvest fruit development of miR164a#STTM, WT and miR164a#OE tomato under control and salt treatment. Changes of color difference L* value (**B**), a* value (**C**), b* value (**D**) of STTM-miR164a, WT and OE-miR164a postharvest tomato fruit at Br to Br + 7 day under salt treatment. Vertical bars represent standard deviations of the means, *n* = 3. Different letters indicated significant differences between WT and transgenic tomatoes in the values at *p* < 0.05.

The color changes of postharvest tomato fruits during ripening under both control and salt stress are shown in Figure 5A. Under control treatments, the miR164a#STTM fruits changed color more rapidly than WT, but miR164a#OE fruits changed color more slowly than WT. After salt stress treatment, postharvest tomato fruits changed color more rapidly than control fruits. Compared with WT, the color of miR164a#STTM fruits changed more rapidly, while the color of miR164a#OE fruits changed more slowly under salt stress. Under salt stress, the miR164a#STTM tomato fruits completely turned red 5–6 days after color breakage, while WT tomato fruits completely turned red only 7 days after color breakage; miR164a#OE tomato fruit did not completely turn red 7 days after breaking. As shown in Figure 5B–D, the color of fruit gradually changes from green to red with the extension of storage time. The L* value gradually decreased, the a* value gradually increased, and the b* value increased first and then decreased. At Br + 7 day, the L* values of miR164a#STTM, WT and miR164a#OE tomato fruits were 34.04, 43.08 and 48.90, the a* values were 34.59, 29.36 and 25.18, the b* values were 42.09, 42.09 and 43.58, respectively.

**Figure 6:**
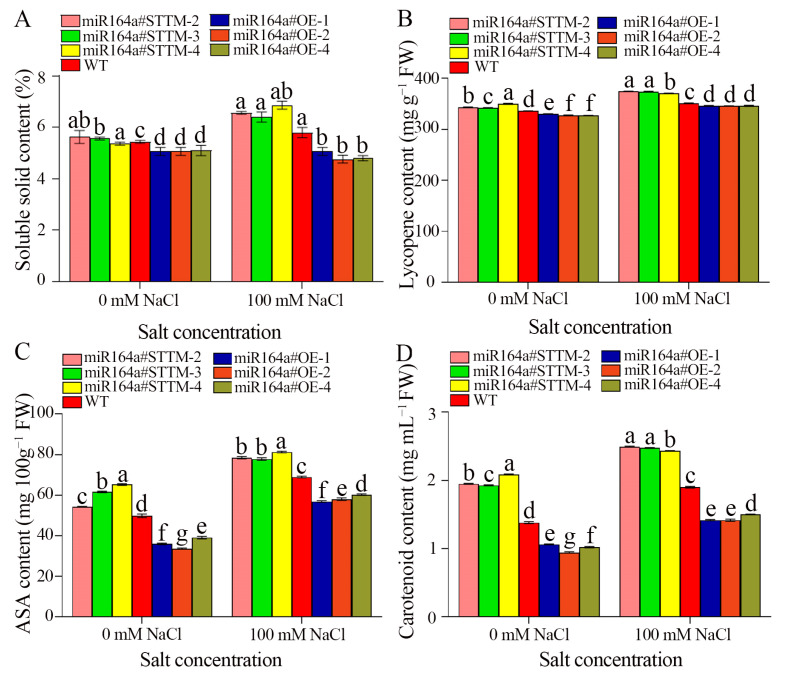
The contents of soluble solids (**A**), lycopene (**B**), ASA (**C**), carotenoids (**D**) in miR164a#STTM, WT and miR164a#OE postharvest tomato fruit during salt treatment. Vertical bars represent standard deviations of the means, *n* = 3. Different letters indicated significant differences between WT and transgenic tomatoes in the values at *p* < 0.05.

Under 100 mM NaCl stress, the average soluble solids content of miR164a#STTM-2, miR164a#STTM-3, miR164a#STTM-4 and WT tomato fruits were 6.57, 6.40, 6.87 and 5.80, respectively. Compared with the control, the soluble solids content of miR164a#STTM-2, miR164a#STTM-3, miR164a#STTM-4 and WT fruit increased by 14.2%, 13.0%, 21.8% and 6.3%, respectively (Figure 6A). The average lycopene content of miR164a#STTM, WT and miR164a#OE tomato fruits were 373.11, 351.38 and 346.05 mg g**^−^**^1^ FW, respectively. The lycopene content of miR164a#STTM fruit was 1.06 times that of WT, and that of miR164a#OE was 0.98 times that of WT (Figure 6B). The average content of ASA in miR164a#STTM, WT and miR164a#OE tomato fruits were 79.14, 68.91 and 58.34 mg 100 g**^−^**^1^ FW, respectively. The ASA content of miR164a#STTM fruit was 1.15 times that of WT, and miR164a#OE was 0.85 times that of WT (Figure 6C). The average carotenoid content in miR164a#STTM, WT and miR164a#OE tomato fruits were 2.47, 1.90 and 1.44 mg mL**^−^**^1^ FW, respectively. The carotenoid content in miR164a#STTM fruit was 1.30 times that of WT, and miR164a#OE was 0.76 times that of WT (Figure 6D).

### 2.5. Knockdown of Sly-miR164a Increased ABA Content under Salt Stress

**Figure 7:**
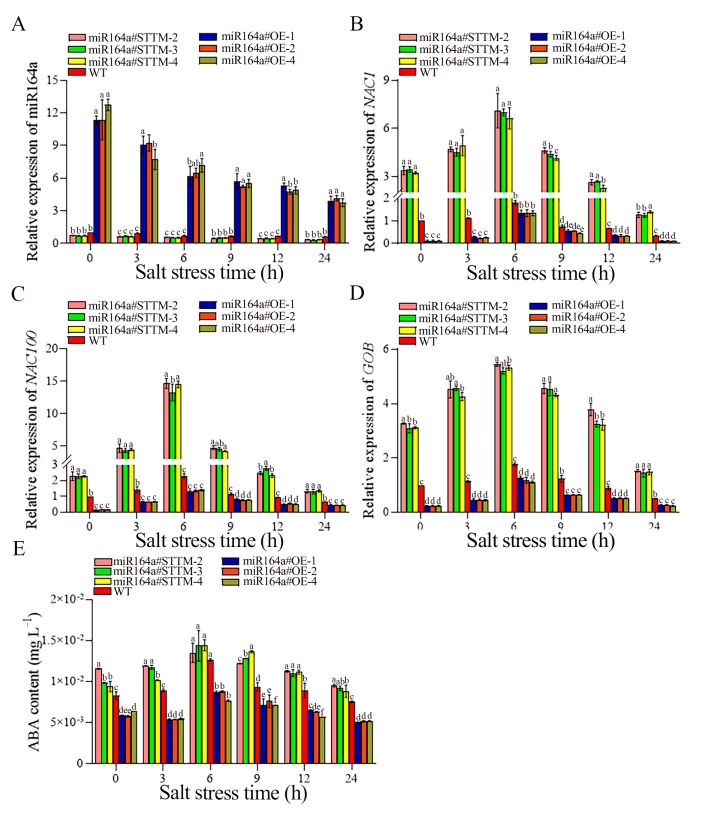
The expression levels of miR164a (**A**), *NAC1* (**B**), *NAC100* (**C**), *GOB* (**D**) and ABA content (**E**) in miR164a#STTM, WT and miR164a#OE tomato seedlings root during salt treatment. Vertical bars represent standard deviations of the means, *n* = 3. Different letters indicated significant differences between WT and transgenic tomatoes in the values at *p* < 0.05.

As shown in Figure 7A, under 100 mM NaCl stress, the relative expression of Sly-miR164a in miR164a#STTM, miR164a#OE and WT tomato roots showed a downward trend. After 24 h of salt stress, the levels of relative expression of Sly-miR164a in miR164a#STTM-2, miR164a#STTM-3, miR164a#STTM-4, WT, miR164a#OE-1, miR164a#OE-2 and miR164a#OE-4 tomato roots were 0.34, 0.33, 0.34, 0.63, 3.89, 4.15 and 3.75, respectively. The relative expression of Sly-miR164a in miR164a#STTM roots was 0.54 times that of WT, while the relative expression of Sly-miR164a in miR164a#OE roots was 6.25 times that of WT. At 6 h of salt stress, the relative expression of *NAC1*, *NAC100* and *GOB* in miR164a#STTM, WT and miR164a#OE tomato roots reached their highest levels, and then gradually decreased (Figure 7B–D). After 6 h of salt stress, the relative expression of *NAC1* in miR164a#STTM-2, miR164a#STTM-3, miR164a#STTM-4, WT, miR164a#OE-1, miR164a#OE-2 and miR164a#OE-4 was 7.10, 7.00, 6.62, 1.78, 1.36, 1.33 and 1.33, respectively. The relative expression of *NAC100* in miR164a#STTM-2, miR164a#STTM-3, miR164a#STTM-4, WT, miR164a#OE-1, miR164a#OE-2 and miR164a#OE-4 was 14.67, 13.27, 14.50, 2.29, 1.31, 1.33 and 1.38, respectively. The relative expression of *GOB* in miR164a#STTM-2, miR164a#STTM-3, miR164a#STTM-4, WT, miR164a#OE-1, miR164a#OE-2 and miR164a#OE-4 was 5.44, 5.20, 5.32, 1.76, 1.26, 1.18 and 1.10, respectively. After 24 h of salt stress, the relative expression of *NAC1* in miR164a#STTM-2, miR164a#STTM-3 and miR164a#STTM-4 was 3.98, 3.93 and 3.71 times of WT, respectively. The relative expression of *NAC100* in miR164a#STTM-2, miR164a#STTM-3 and miR164a#STTM-4 was 6.40, 5.79 and 6.33 times of WT, respectively. The relative expression of *GOB* in miR164a#STTM-2, miR164a#STTM-3 and miR164a#STTM-4 was 3.09, 2.95 and 3.02 times of WT, respectively. Under salt treatment, the accumulation of ABA in miR164a#STTM transgenic lines was higher than that in WT, while that in miR164a#OE transgenic lines was lower than that in WT. After 24 h of salt stress, the ABA content in miR164a#STTM-2, miR164a#STTM-3 and miR164a#STTM-4 tomato roots was 1.26, 1.21 and 1.17 times that of WT, and the ABA content in miR164a#OE-1, miR164a#OE-2 and miR164a#OE-4 tomato roots was 0.66, 0.68 and 0.68 times that of WT (Figure 7E).

## 3. Discussion

Crop development and productivity are greatly hampered by salt stress, which results in significant financial loss. As a critical participant in the response of plants to abiotic stresses, miRNA may improve plant tolerance to these stresses in the process of adapting to adverse environmental conditions [23,24,25]. In maize, cotton and rice plants, some miRNAs have been observed to be overexpressed or silenced to increase plant resistance to various stresses [14,26,27]. The miR164 is a vital plant regulatory factor which is involved in a number of biological processes and is crucial for plant development, growth and stress resistance [28,29]. Previous investigations in wheat found that miR164 reduced wheat seedling tolerance to salt stress by down-regulating the expression of *TaNAC14* [30]; however, the salt stress resistance function of miR164 in tomatoes remains unknown. In this study, transgenic plants were obtained using overexpression and the STTM (Short Tandem Target Mimic) methods to analyze the role of miR164a in regulating tomato salt stress, and its impact on improving tomato salt stress tolerance was further examined. The results showed that the overexpression of miR164a tomato lines decreased tolerance to salt stress, while knockdown of Sly-miR164a with STTM enhanced plant salt tolerance. Under salt stress, Sly-miR164a knockdown in tomatoes enhanced fresh weight, stem diameter, root length, and plant height in comparison to WT.

Salinity stress will induce osmotic stress, ion toxicity and oxidative stress in plants, which will lead to excessive accumulation of toxic compounds and ROS in plants, resulting in peroxidative damage to cell membranes [31,32]. In plant cells, oxidative stress leads to the buildup of ROS like H_2_O_2_ and O^2•−^ and damages the structure of the cell membrane [33]. Under salt stress, the accumulation of MDA in plants increased, which also caused severe membrane lipid oxidative damage [34]. Both are often used to assess plant tolerance to salt. In order to maintain normal metabolic activities and better cope with the environment of salt stress, plants will accumulate some osmotic adjustment substances to alleviate the damage caused by salt stress and enhance plant salt stress tolerance [35]. Proline is an effective organic compound involved in plant stress resistance, which can play a role in relieving plant salt stress [36]. In the present study, H_2_O_2_ and O^2•–^ and MDA content in tomato root tissue significantly increased under salt stress. The miR164a#OE lines accumulated more H_2_O_2_ and O^2•–^ content than WT, while miR164a#STTM lines accumulated less H_2_O_2_ and O^2•–^ content (Figure 2B,C). Compared with WT, the MDA content in miR164a#STTM tomato plants was reduced, while the MDA content in miR164a#OE transgenic plants was significantly increased (Figure 2D). The proline content of the miR164a#STTM strain was significantly higher than that of WT and miR164a#OE (Figure 2E). The results indicated that miR164a#STTM plants enhanced the salt tolerance by increasing the content of proline and maintaining the integrity of the cell membrane of miR164a#STTM tomato plants. 

Reduced fruit water and an accumulation of solute activity under salt stress resulted in a rise in soluble solids content, which enhanced fruit quality [37]. Li et al. [38] found that moderate salt treatments (20 and 60 mM) increased fresh weight, soluble solids and anthocyanin content and improved overall fruit quality, while high salinity treatments (100 and 150 mM) decreased fruit quality in grape berries. The quality of strawberry fruit was improved by salt stress, which decreased the fruit’s average weight and water content while increasing its ion, ascorbic acid, and soluble solids contents [39]. Moderate saline irrigation increased the content of carotenoids, lycopene and ascorbic acid in tomato fruits, so tomato fruits had higher antioxidant capacity [40]. Massaretto et al. [41] showed that salt stress treatment reduced the yield and fruit size of tomato fruits, but increased the content of carotenoid and soluble solids in fruits, which gave tomato fruits higher antioxidant capacity and improved fruit quality. In addition, vitamin C content in fruit decreased with the increase of salt stress concentration [42]. In the present results, under salt stress, the contents of soluble solids, lycopene, ASA and carotenoid in miR164a#STTM fruits are more than WT, and the fruit quality is superior to WT. The results of previous studies also showed that under salt stress, the yield and quality of salt-tolerant tomato plants were less affected by salt stress, and the accumulation of soluble solids and soluble sugars in salt-tolerant tomato fruits increased, which is consistent with our findings [43]. The results indicated that the improvement of miR164a#STTM fruit quality may be related to the enhancement of salt tolerance of tomato by knockdown of Sly-miR164a. The miRNAs exert an essential action in regulating plant responses to various abiotic stresses at the level of transcription factors that regulate the expression of genes associated with abiotic stress responses and can activate and repress various plant defense pathways [44]. The miR164 is a large family of plant-specific genes that target NAC transcription factors [45]. One of the largest groups of transcriptional regulators is the NAC transcription factor family [46]. Previous studies have found that overexpressing the NAC transcription factor in tomato boosted the accumulation of stress-induced proline and lowered the accumulation of reactive oxygen species, which helped the plant become more tolerant to abiotic stress [47]. Compared with the wild type, the overexpression of *OsNAC5* in rice increased stress resistance, enlarged rhizomes, and increased grain yield [48]. Overexpression of cotton *SNAC1* improved cotton’s tolerance to salt [49]. Abiotic stress can be directly responded to by the NAC transcription factor family through ABA independent and ABA dependent mechanisms [50]. ABA is an essential phytohormone with a significant function in regulating plant responses to stress [51]. Increased osmotic pressure from salt stress will lower soil water potential, encourage an increase in ABA content, and be crucial in reducing the stress response [52,53]. Under long-term salt stress, stomatal closure and increased proline content were induced by an ABA-dependent pathway to reduce stress [54]. Under mild salt stress, ABA metabolism in strawberries is affected and results in an increase in ABA content, phenols, anthocyanins and ascorbic acid content, as well as an increase in antioxidant activity [55]. The rice stress-responsive NAC gene (*ONAC022*) increases endogenous ABA, proline, and soluble sugar content by modulating ABA-mediated pathways, and the salt tolerance of transgenic plants is enhanced [56]. The expression of *NAC1*, *NAC100*, and *GOB* was significantly increased in tomato roots of miR164a#STTM under salt stress (Figure 7B–D), and the ABA level was higher than that of WT during salt stress (Figure 7E). Therefore, knockdown of Sly-miR164 in tomato may also improve salt resistance by regulating ABA levels.

## 4. Materials and Methods

### 4.1. Plant Vector Construction and Transformation

The mature sequence of tomato Sly-miR164a was derived from miRBase database (http://www.mirbase.org/) accessed on 5 March 2021. To generate Sly-miR164a-knockdown tomato plants, the short tandem target mimic (STTM) was designed as previously described by Yan et al. [57], and connected to the pBWA(V)HS vector (Supplementary File S1). The pBI121-miR164a overexpression vector was created using the method described by Zhao et al. [58] (Supplementary File S2). The vector was transformed into the *Agrobacterium tumefaciens* strain GV3101 for tomato transformation after being sequence verified.

### 4.2. Plant Material and Salt Treatment

For this experiment, Zhiqiang Wang kindly provided T3 homozygous transgenic seeds of miR164a#STTM and miR164a#OE and WT seeds [59] (miR164a#STTM-2 represents STTM-miR164a#2-18-4; miR164a#STTM-3 represents STTM-miR164a#5-20-11; miR164a#STTM-4 represents STTM-miR164a#7-21-14; miR164a#OE-1 represents OE-miR164a#13-11-1; miR164a#OE-2 represents OE-miR164a#15-12-15; miR164a#OE-4 represents OE-miR164a#16-13-19). Surface sterilization with 3% sodium hypochlorite was performed on the seeds of miR164a#STTM, miR164a#OE and WT for 5 min. The seeds were sterilized before being placed on a ½ × MS agar medium containing a 50 mM NaCl concentration and cultivated vertically for 14 days. The root lengths were measured using a centimeter ruler after 14 days of vertical culture, and all seedlings were weighed on an analytical balance to assess their growth status. At least 10 seeds were analyzed at a time and the experiment was repeated three times.

The WT and miR164a#STTM and miR164a#OE transgenic lines seeds were planted in opaque pots with a soil mix of peat soil, laterite and vermiculite (2.2:1:0.8), and the plants were grown in a greenhouse at 25 °C, 16 h of light, and 8 h of dark light. Three-week-old (tomato seedlings with 4 to 5 true leaves are fully unfolded) WT, miR164a#STTM and miR164a#OE tomato seedlings were treated with salt stress by irrigating them with 100 mM NaCl every 4 days; the control group was irrigated with water. The plant height and stem diameter were measured and photographed to record the phenotype at 4, 8, 12, 16, 20, and 24 days after treatment initiation. A ruler was used to measure the height of the plant from its base to its highest point. The stem diameter of the tomato was measured using a vernier caliper scale at a height of 2 cm from the ground. After being exposed to salt stress for 0, 7 and 14 days, all the roots from five WT and miR164a#STTM and miR164a#OE transgenic lines were cleaned with sterile water after being removed from the soil, then frozen in the liquid nitrogen, and kept at −80 °C. For RNA analysis, the roots that had been exposed to 100 mM NaCl were taken at 0, 3, 6, 9, and 12 h. Each treatment contained 5 seedlings and three replicates were performed per experiment.

The control fruits were harvested from tomato plants that had not been treated with water for 60 days, and the salt-stress fruits were harvested from tomato plants that had been treated with salt stress for 60 days. At least 18 ripening stage (BR) tomato fruits were selected from 6 different WT and miR164a#OE and miR164a#STTM transgenic lines, with 6 healthy and uniform tomato fruits selected from each plant. In transgenic miR164a#OE and miR164a#STTM and WT lines, 18 fruits at the breaking (Br) stage were similarly picked and kept at room temperature until Br + 7 days. Tomato fruits of preharvest and postharvest were photographed from the day of color break (BR days) to the 8th (Br + 7 days) and color changes were recorded. For the samples of preharvest fruits and postharvest fruits at the ripening stage (Br + 7 days), seeds were removed and fruit peel tissue were retained, ground to a powder with liquid nitrogen and stored at −80 °C and physiological data were collected.

### 4.3. Histochemical Staining of H_2_O_2_ and O^2•–^

The first leaf of the third branch of three-week-old seedlings was stained with NBT solution (1 mg mL^−1^ in 10 mM phosphate buffer; pH 7.8) overnight in the dark after they had been exposed to salt (100 mM NaCl) for 14 days. The leaf was left to soak in a 1 mg mL^−1^ solution of DAB (pH 3.8) in the dark overnight. To remove chlorophyll, the discolored leaves were boiled in 95% ethanol for 10 min. Following staining, the color shades of the miR164a#STTM, WT, and miR164a#OE leaves’ NBT and DAB staining were assessed through visual inspection.

### 4.4. Quantification of H_2_O_2_, O^2•–^, MDA and Proline Content

Three-week-old tomato seedlings of WT, miR164a#STTM and miR164a#OE were cultivated in plates containing 100 mM NaCl solution for 14 days, while control tomato seedlings were placed in plates containing water. Tomato seedling roots were taken at 0, 7 and 14 days and stored at −80 °C. The content of H_2_O_2_ and O^2•–^ was measured according to the method of the Assay Kit (Solarbio Inc., Beijing, China). The H_2_O_2_ and O^2•–^ content both were expressed as μmol g^−1^ FW. The method of Wang et al. [60] was used to determine the MDA content. The approach of Ge et al. [61] was used to identify the proline content.

### 4.5. Fruit Color

Three fruits at the ripening stage (BR) were selected in each transgenic miR164a#OE and miR164a#STTM and WT lines and stored at room temperature until Br + 7 days. The color of tomato peel was measured at three positions in the middle of the fruit using the color difference meter (CR-400, Konica Minolta Camera Co., Ltd., Osaka, Japan) from the day of color breakage (BR day) to the 8th (BR + 7 days), and L*, a* and b* values were recorded.

### 4.6. The Soluble Solid, Lycopene, Ascorbic Acid and Carotenoid Content of Tomato Fruit

The content of soluble solids in tomato fruit was determined by saccharometer (H1987, Deke Machinery Technology Co., Ltd., Xingtai, China) according to the method Duan et al. [62]. The lycopene content was determined according to the method described by Fish et al. [63], with slight modifications. Briefly, ground tomato peel tissue (0.1 g) was weighed and homogenized in hexane-acetone-ethanol (ratio 2:1:1, *v*/*v*/*v*). After homogenization, 0.3 mL of distilled water was added and the mixture was immediately placed on ice for 5 min for better phase separation. After separation, the absorbance of the supernatant containing lycopene (hexane) was measured at 503 nm. The concentration of lycopene was estimated by the following equation:

Lycopene (mg g^−1^) = 31.2 × (OD_503_)/g FW

The content of ASA was determined by the method of Zhang et al. [64] with some modification, and expressed as mg Kg^−1^ FW. Briefly, tomato peel tissues (0.2 g) were homogenized in 2 mL of 5% trichloroacetic acid (TCA). The homogenate was then centrifuged at 12,000× *g* and 4 °C for 15 min. The 1.0 mL extracted supernatant was mixed with 1.0 mL of 50 g L^−1^ TCA solution, 1.0 mL of absolute ethyl alcohol, 0.5 mL of 0.4% phosphoric acid-ethyl alcohol, 1.0 mL of 5 g L^−1^ red phenanthroline-ethyl alcohol and 0.5 mL of 0.3 g L^−1^ ferric chloride-ethyl alcohol. The absorbance of the supernatant was measured at 534 nm using a microplate reader (SpectraMax190; Molecular Devices, Sunnyvale, CA, USA). The content of carotenoids was determined by the method of Zhu et al. [65], and expressed as mg mL^−1^ FW.

### 4.7. Determination of ABA Content

The ELISA Kit (enzyme label Biotechnology Co., Ltd., Yancheng, China) was used to measure the content of ABA in the sample. At 450 nm, the content of ABA in the root tissue was measured. The content of ABA was given as mg L^−1^.

### 4.8. RNA Extraction and Quantitative Real-Time PCR Analysis

RNA was extracted from root tissues with the Fast Pure Plant Total RNA Kit (Tiangen Biotechnology Co., Ltd., Ltd., Nanjing, China). The samples’ RNA integrity was examined using agarose gel electrophoresis. The relative expression of Sly-miR164a, *NAC1*, *NAC100* and *GOB* was determined by qRT-PCR. The miR164a and target genes (*NAC1*, *NAC100* and *GOB*) quantifications were performed with the *U6* and *Actin* as the internal reference gene, respectively. The relative expression levels were calculated using the equation 2^-ΔΔCT^. Primer sequences used for qRT-PCR are listed in Supplementary Table S1.

### 4.9. Statistical Analysis

The experimental results were analyzed using a one-way ANOVA (*p* < 0.05) in IBM SPSS Statistics 23.0 (SPSS, Chicago, IL, USA), and different letters indicated significant difference between WT and transgenic tomatoes. The results are presented as the mean and standard deviation of three replications.

## 5. Conclusions

Overexpression of Sly-miR164a increased the sensitivity of tomato plants to salt. Knockdown of the Sly-miR164 in tomato plants played a positive regulatory role in response to salt stress, which enhanced the expression of *NAC* and decreased ROS and MDA accumulation levels in transgenic plants, and improved the preharvest and postharvest fruit nutritional value of tomato.

## Data Availability

Not applicable.

## Supplementary Materials

The following supporting information can be downloaded at: https://www.mdpi.com/article/10.3390/ijms24054639/s1.
