# Supplementary material for: Knockdown of Sly-miR164a Enhanced Plant Salt Tolerance and Improved Preharvest and Postharvest Fruit Nutrition of Tomato
Source: Int J Mol Sci. 2023 Feb 27;24(5):4639. doi: 10.3390/ijms24054639 (PMC10003209; doi:10.3390/ijms24054639)
Supplement: Supplementary file 1 [file ijms-24-04639-s001.zip › Supplementary File S1.pdf]

Supplementary File S1

The sequences of STTM 164a. Yellow shades indicate the 48 nt spacer. Green shades indicate the STTM 164a sequences. Blue shades and Bold indicate the *BsaI* and *Eco31I* enzyme digestion sites, respectively. Blue shades indicate the 2 ×35PS and T35PS, respectively.

>STTM 164a

CTTCAAAGCAAGTGGATTGATGTGATATCTCCACTGACGTAAGGGATGACG

CACAATCCCACTATCCTTCGCAAGACCCTTCCTCTATATAAGGAAGTTCATT

TCATTTGGAGAGAACACGGGGGACTTTGA**GGCAAC**TGCACGTGCCCCTAT

GCTTCTCCAGTTGTTGTTGTTATGGTCTAATTTAAATATGGTCTAAAGAAGA

AGAATTGCACGTGCCCCTATGCTTCTCCA**TGTAAC**TAGCTCTGTCTTCAGTA

CTGGGCCCGAAGACTGACCAGCTCGAATTCCCCGATCGTCAAACATTGCAA

TA AGTA
