# Supplementary material for: Knockdown of Sly-miR164a Enhanced Plant Salt Tolerance and Improved Preharvest and Postharvest Fruit Nutrition of Tomato
Source: Int J Mol Sci. 2023 Feb 27;24(5):4639. doi: 10.3390/ijms24054639 (PMC10003209; doi:10.3390/ijms24054639)
Supplement: Supplementary file 1 [file ijms-24-04639-s001.zip › Supplementary File S2.pdf]

Supplementary File S2

The sequences of pri-miR164a. Yellow shades indicate the precursor sequence of miR164a. Green shades and bold indicate the miR164a stem-loop sequence and the mature miR164a sequences, respectively.

>pri-miR164a

CTACCACATCCTCACATCTTTTTCCAGCTATAAATACTTGAGCTACCCCAATC

TTCTTATTTGTCACCTCAAGTTCAAAAATGAACAATTCACTCACACAATGCA

AGAAAATACCTTTTTTCTTTTTTTAAAGTTGTAGTTAACTTTAATGGAAATGT

TGGCAATAATGGGTGGGTAACTCATGT**TGGAGAAGCAGGGCACGTGCAA**

ATTCTTGTATTCGACAATATATGCATTACTAATGGTAATGCATGAATTTTGCA

CGTGTTCTCCTTCTCCAACCCGAATTTCCTACCTCCTCTCTTCTCAAGATTCT

TCAAAATTCAAACCACTTTTGACCTAATGTGCCTTGAGACGTAATTGTTGTA

CATTACTTTTTTAAAGTATACGCTATAATACCAAGGAAACATTAACGACTAA

ACTAATGGCATGCATGACTATGCCCTTGACTAAA
