# Supplementary material for: Knockdown of Sly-miR164a Enhanced Plant Salt Tolerance and Improved Preharvest and Postharvest Fruit Nutrition of Tomato
Source: Int J Mol Sci. 2023 Feb 27;24(5):4639. doi: 10.3390/ijms24054639 (PMC10003209; doi:10.3390/ijms24054639)
Supplement: Supplementary file 1 [file ijms-24-04639-s001.zip › Supplementary Table S1.pdf]

Supplementary Table S1

Table S1. List of primers sequences.

**Gene and miRNA**	**primer**	**Sequence (5'-3')**
SlNAC1	Forward Primer	CGACCAAACAAACCCTAACAAC
	Reverse Primer	TGGTTAGGGGTGAAAATGGAG
SlNAC100	Forward Primer	ACTGCTACTGCTTCGAAATCCA
	Reverse Primer	TGAATGGAGCTATTTGGTTACAAGA
GOB	Forward Primer	TCGATTCCTCTCCGTATAGCAC
	Reverse Primer	GTCGAAGACAGAAGTTGGATCG
RT-miR164a	Forward Primer	GTCGTATCCAGTGCAGGGTC
	Reverse Primer	CGAGGTATTCGCACTGGATACGACTGCACG
Sly-miR164a	Forward Primer	GCGGCGGTGGAGAAGCAGGGCA
	Reverse Primer	GTGCAGGGTCCGAGGT
TOM-U6	Forward Primer	TCTAACAGTGTAGTTTGTCCCTTCG
	Reverse Primer	TTGTGCGTGTCATCCTTGC
SlActin	Forward Primer	ATCCACGAGACTACCTACA
	Reverse Primer	CTCATACGGTCAGCAATA
